# Supplementary material for: Elevated METTL9 is associated with peritoneal dissemination in human scirrhous gastric cancers
Source: Biochem Biophys Rep. 2022 Apr 2;30:101255. doi: 10.1016/j.bbrep.2022.101255 (PMC8983939; doi:10.1016/j.bbrep.2022.101255)
Supplement: Multimedia component 1 [file mmc1.pdf]

Supplementary Materials

Elevated METTL9 is associated with peritoneal dissemination in human scirrhous gastric cancers

Toshifumi Hara, Yuuki Tominaga, Koji Ueda, Keichiro Mihara, Kazuyoshi Yanagihara, Yoshifumi Takei

Contents:

Supplementary Fig. 1
Supplementary Figure Legend
Supplementary Methods
Supplementary Table 1
Supplementary Table 2

Supplementary Fig. 1

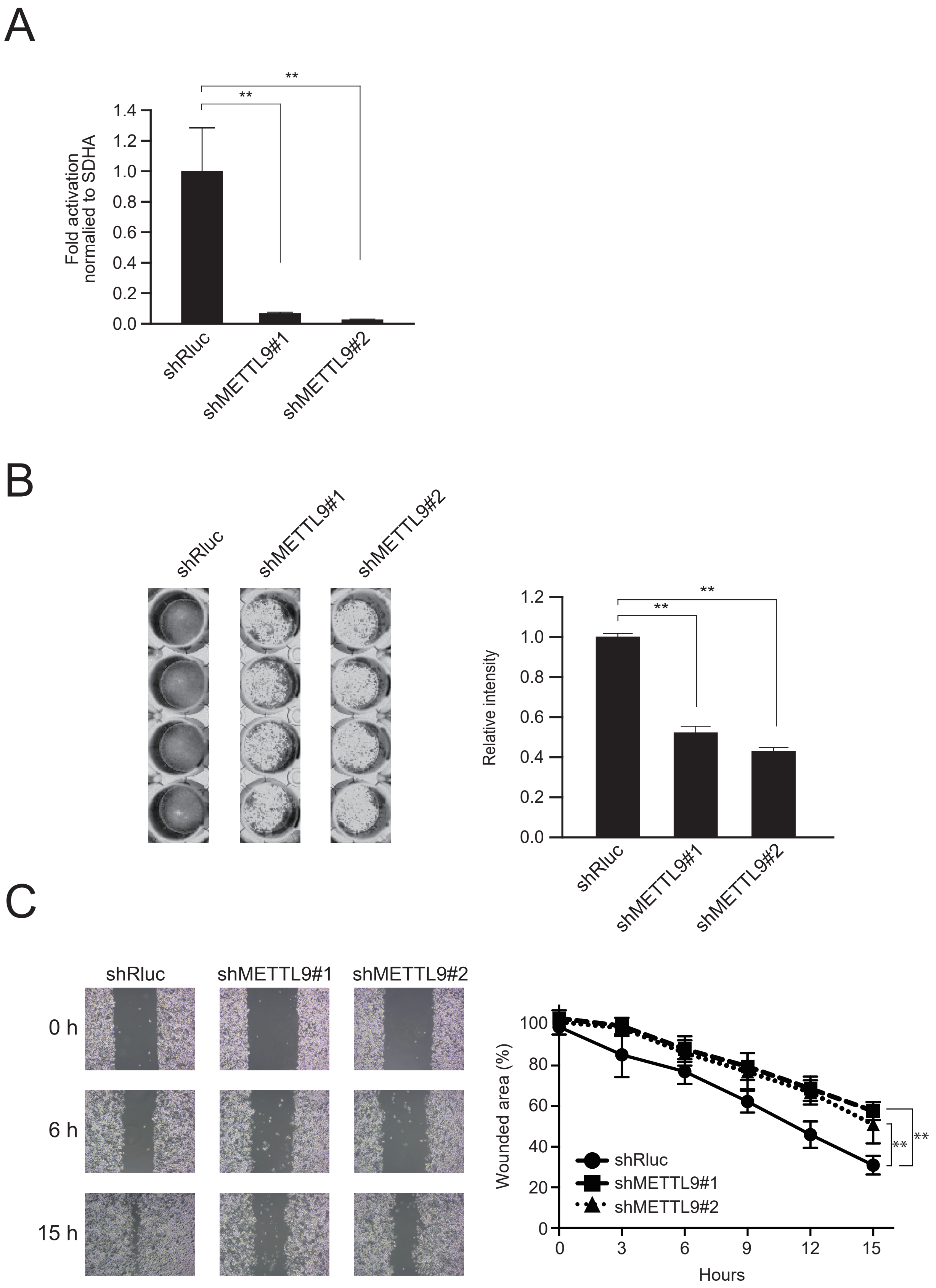

Supplementary Figure Legend

Supplementary Fig 1.

Similar phenotypic effects were obtained in METTL9-knockdown in 44As3 metastatic cells established from another scirrhous gastric cancer patient.

(A) Knockdown efficiencies of shRNA targeting METTL9 in 44As3 were examined by RT-qPCR. Both shMETTL9#1 and shMETTL9#2 showed significant reductions in METTL9 expression compared with shRluc. **, $p<0.01$.

(B) Clonogenic assay. After crystal violet staining, the images were obtained. The pixel intensity of each image was analyzed by Image J software, as shown in the right panel in (B). Significantly fewer colonies of METTL9 knockdown 44As3 cells were formed compared with the control cells (shRluc). **, $p<0.01$.

(C) Cell migration assay (scratch assay). The effect of METTL9 knockdown 44As3 metastatic cells on cell migration was examined by the wound-healing assay method. Knockdown of METTL9 in 44As3 cells revealed significant inhibition of cell migration compared with the control cells (shRluc). **, $p<0.01$.

Supplementary Methods

Clonogenic assay

Five-hundred cells were seeded in a 24-well plate. After 10 days, the cells were fixed with ice-cold methanol for 10 minutes and then stained with 0.5% crystal violet solution containing 20% methanol for 30 min. The cells were washed with distilled water 3 times and placed at room temperature until completely dried. The picture was taken with an LAS 4000 digital imaging system (GE Healthcare).

Wound-healing assay

Cells (5×10^5) were seeded in a culture insert (iBidi) placed onto a 24-well plate. After 24 hours, the culture insert was removed and then a picture of the wounded areas was taken through a microscope every 3 hours. The wounded region was calculated by ImageJ software with the MRI Wound Healing Tool.

Supplementary Table 1. Target sequences of shRNAs for human METTL9.

**shRNAs**	**Target sequences (5' -> 3')**
shMETTL9#1	TTAAGTATAAAAAATATCTTCC
shMETTL9#2	TTAAATTCCTACATGATATTTA

Technical note:

Potential sequences of shRNA targeting METTL9 were selected according to the splashRNA website as described in Material and Methods. Oligonucleotides containing the target sequences were constructed with XhoI/EcoRI sites in the SGEP vector. The inserted nucleotide sequences of all vectors were analyzed by DNA sequencing.

Supplementary Table 2. Specific primer sequences used for RT-qPCR.

**Primers**	**Sequences (5’ -> 3’)**
METTL9 forward	CTGTGATCAGCCCCTGACTT
METTL9 reverse	TGATGGTTTCTCCCACTTGCC
SDHA forward	TGGGAACAAGAGGGCATCTG
SDHA reverse	CCACCACTGCATCAAATTCATG

Technical note:

Technical note:

The primer set for RT-qPCR was designed to span exon junctions of the target gene and synthesized by Integrated DNA Technologies (IDT) as described in Material and Methods. All reagents were mixed on ice to suppress nonspecific gene amplification. After qPCR, a unique peak was confirmed in a melting curve analysis, indicating a single and specific product generated by PCR.
