# Supplementary material for: Elevated METTL9 is associated with peritoneal dissemination in human scirrhous gastric cancers
Source: Biochem Biophys Rep. 2022 Apr 2;30:101255. doi: 10.1016/j.bbrep.2022.101255 (PMC8983939; doi:10.1016/j.bbrep.2022.101255)

Three repeats of original immunoblots shown in Fig. 1

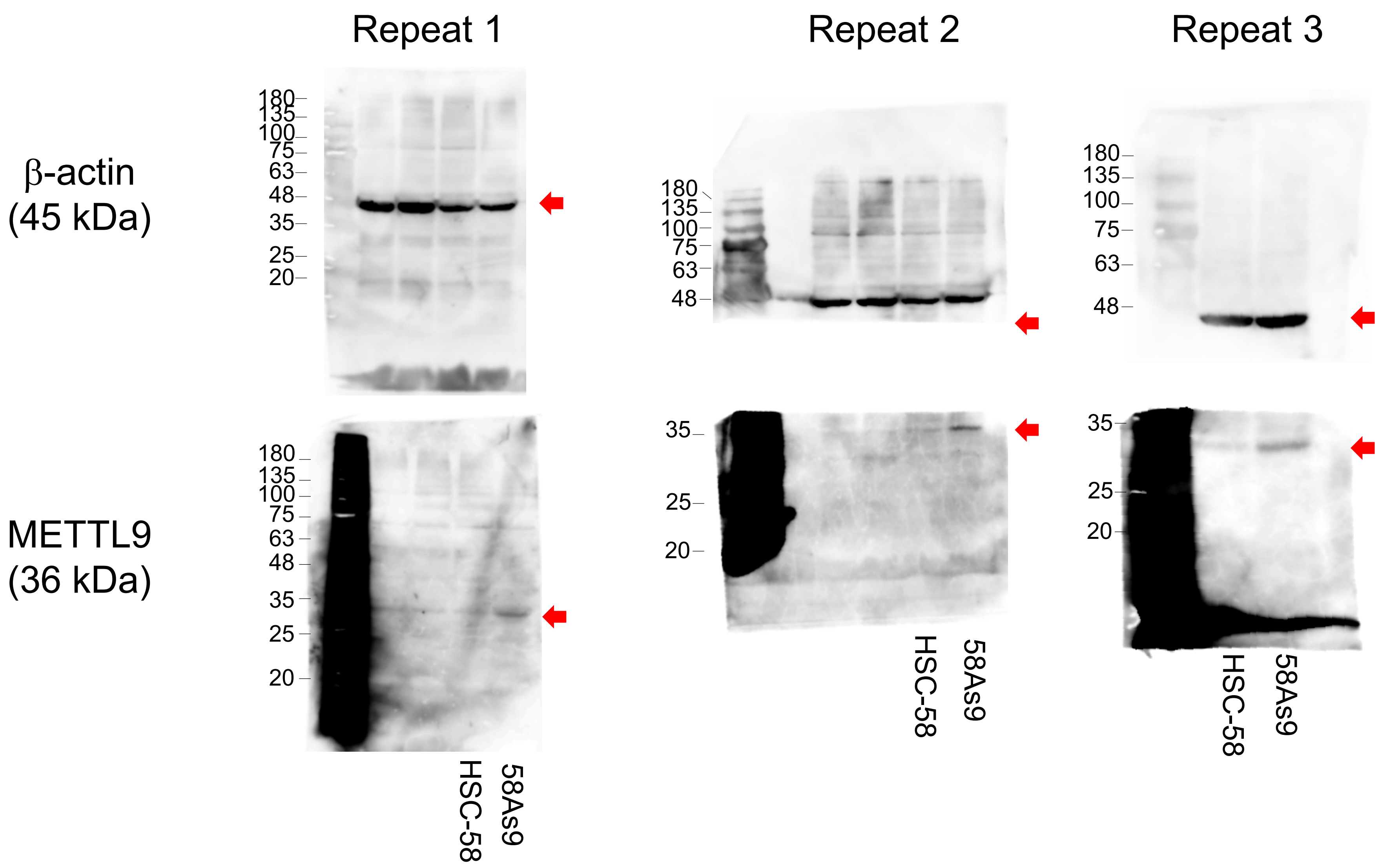

The size of the detected METTL9 band was lower than the expected/calculated size of METTL9. However, the results of the METTL9 knockdown shown in Fig 2 indicate that this band is METTL9.

Three repeats of original immunoblots shown in Fig. 2

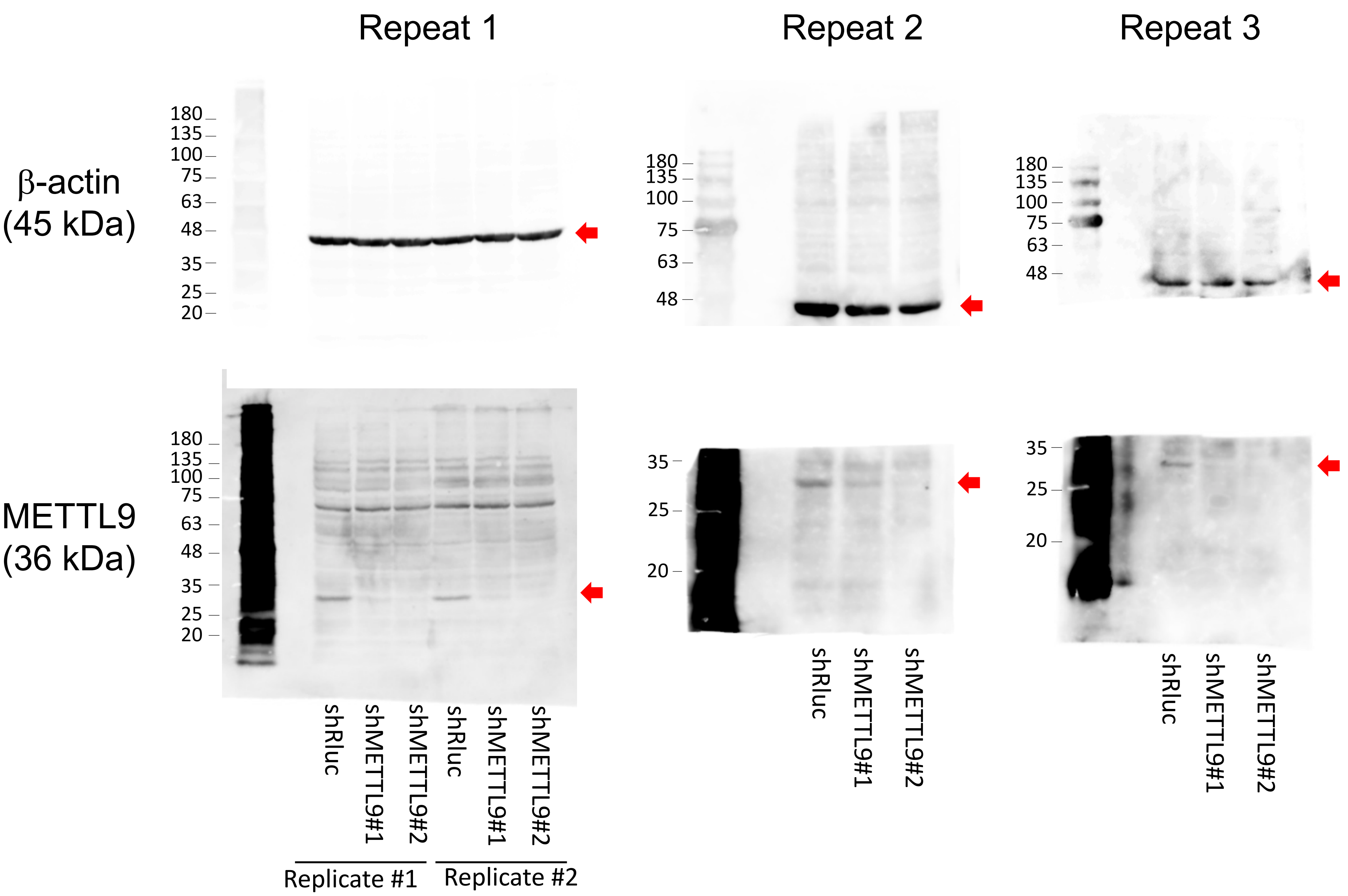

Supplement: Multimedia component 2 [file mmc2.pdf]
